# Postmortem imaging in goats using computed tomography with air as a negative contrast agent

**DOI:** 10.1371/journal.pone.0215758

**Published:** 2019-04-23

**Authors:** Olga Szaluś-Jordanow, Joanna Bonecka, Filip Pankowski, Karolina Barszcz, Sylwia Tarka, Magdalena Kwiatkowska, Michał Polguj, Marcin Mickiewicz, Agata Moroz, Michał Czopowicz, Tadeusz Frymus, Jarosław Kaba

**Affiliations:** 1 Department of Small Animal Diseases with Clinic, Faculty of Veterinary Medicine, Warsaw University of Life Sciences-SGGW, Warsaw, Poland; 2 Department of Morphological Sciences, Faculty of Veterinary Medicine, Warsaw University of Life Sciences-SGGW, Warsaw, Poland; 3 Department of Forensic Medicine, Medical University of Warsaw, Warsaw, Poland; 4 Department of Angiology, Medical University of Łódź, Łódź, Poland; 5 Laboratory of Veterinary Epidemiology and Economics, Warsaw University of Life Sciences-SGGW, Warsaw, Poland; Universidad Francisco de Vitoria, SPAIN

## Abstract

**Purpose:**

Evaluation of the usefulness of air as a negative contrast medium of blood vessels in goats in post mortem computed tomography (PMCT) and establishing the protocol with appropriate doses and timing of the contrast medium administration.

**Methods:**

Thirty three goats were euthanized 10 to 300 min before the study. First, in 3 goats air was administered into the left or right common carotid artery at dose of 60, 100 and 120 ml/kg, and after each dose PMCT was performed in lateral recumbency. As the latter dose proved to visualize blood vessels best, following 30 goats were examined in the same manner but only with the use of air dose of 120 ml/kg. The quality of CT scans was evaluated independently by two board-certified radiologists.

**Results:**

In all studied animals the vascular system filled with air was clearly visualized on CT scans. In most of goats this amount of air revealed vessels smaller than 4 mm in diameter.

**Conclusions:**

PMCT with air as a negative contrast agent may be an alternative technique used in post-mortem angiography.

## Introduction

Post mortem computed tomography (PMCT) is commonly used in both human and veterinary forensic medicine[1–10]. Plain CT examination, without intravenous contrast agent, provides much less information about the vascular system compared to contrast-enhanced examination.

To make the research more accurate, special contrast agent designed for cadavers can be used, however high price and special equipment is their main drawback. Few articles have so far described the use of air as a negative contrast medium.

In recent years a substantial increase in the number of court proceedings regarding animal offenses has been observed. It is believed that, like in humans, imaging techniques for evaluating the cause of animal death will soon become a routine practice.

In human medicine computed tomography has become an important diagnostic method which started to be a standard part of the post-mortem examination. A new field of forensic medicine–forensic radiology–has recently emerged [2].

This modality gives additional information and both methods together with an anatomopathology examination complement each other. The aim of this study was to evaluate the usefulness of air as a negative contrast medium of blood vessels in goats in post mortem computed tomography (PMCT) and to establish the protocol with appropriate doses and timing of the contrast medium administration, so that the technique was repeatable.

## Material and methods

### Animals

The study was performed on 33 adult Polish White Improved and Polish Fawn Improved goats, intended for culling mainly due to emaciation, low milk yield or progressive arthritis. Their age ranged from 1 to 12 years with the median of 4 years (IQR from 2 to 7 years) and body weight from 20 to 54 kg. The study complied with the Directive 2010/63/EU and the Act of Polish Parliament of 15 January 2015 on protection of animals used for scientific purposes (Journal of Laws 2015, item 266). The animals were euthanized under general anesthesia (10mg/kg ketamine + 0.05mg/kg xylazine) by overdosing barbiturates (pentobarbital 30 mg/kg).

### Air administration method

**Fig 1:**
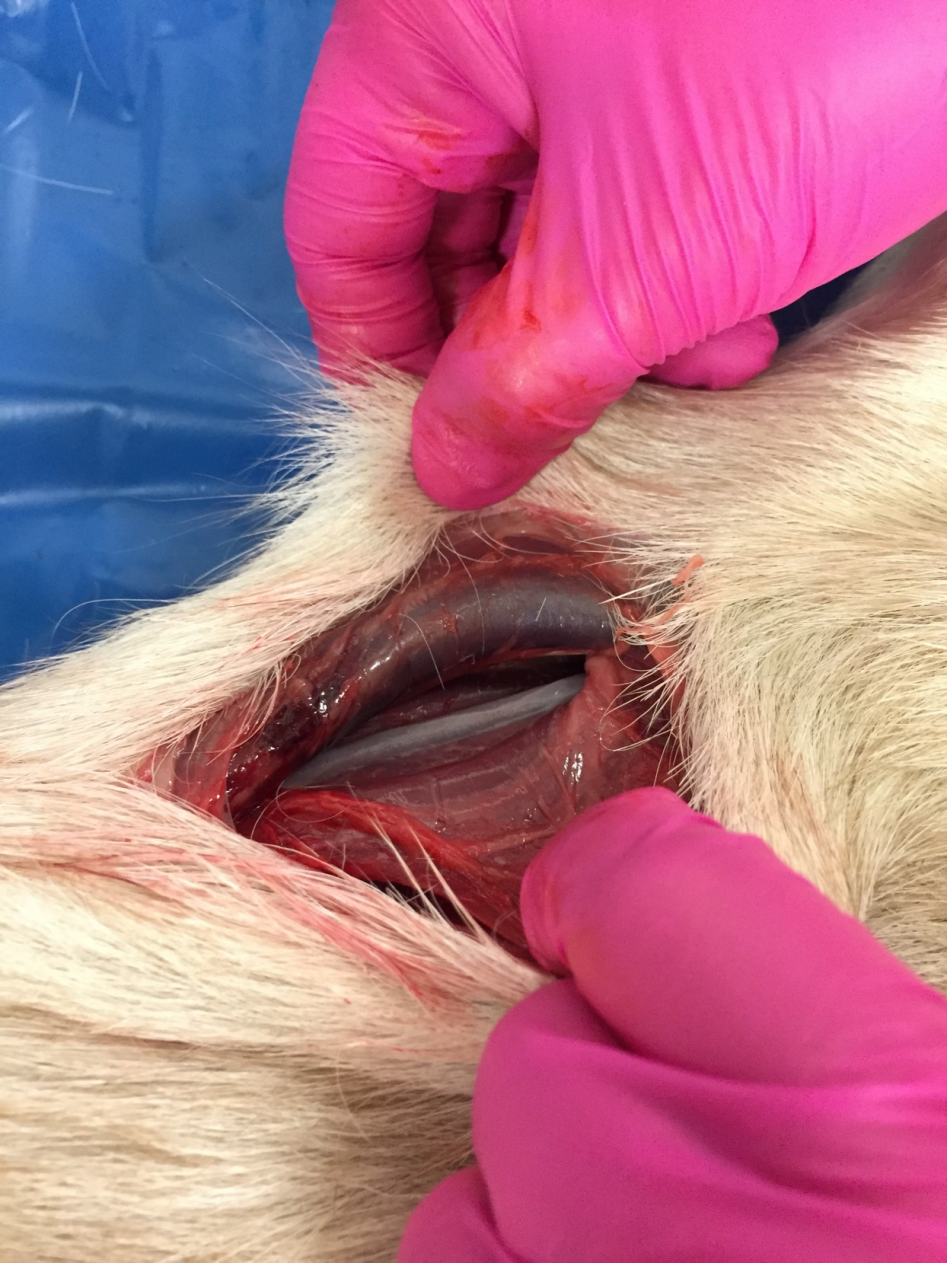
The common carotid artery after cutting the skin and subcutaneous tissues.

**Fig 2:**
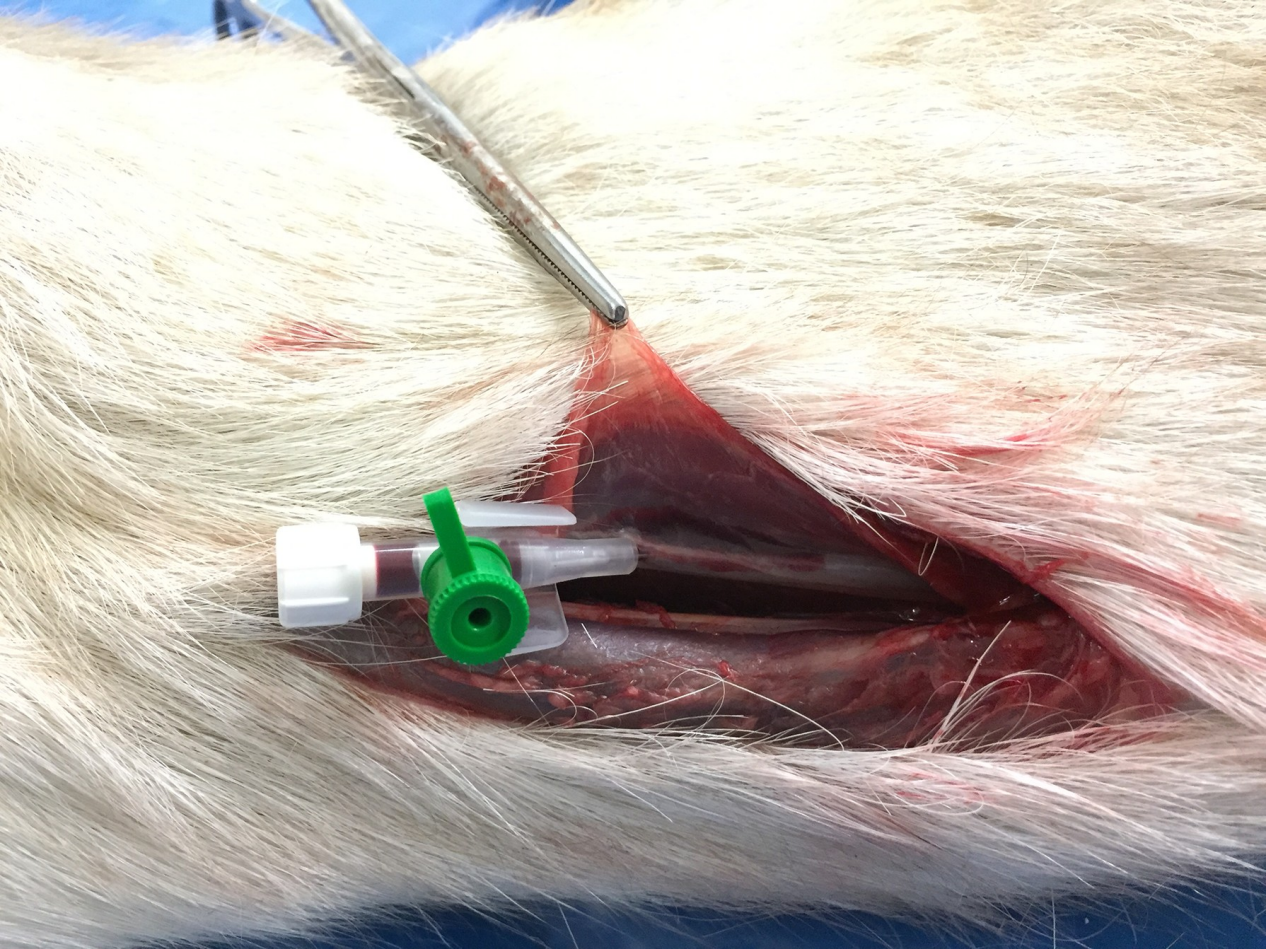
Intravenous catheter placed into the common carotid artery.

**Fig 3:**
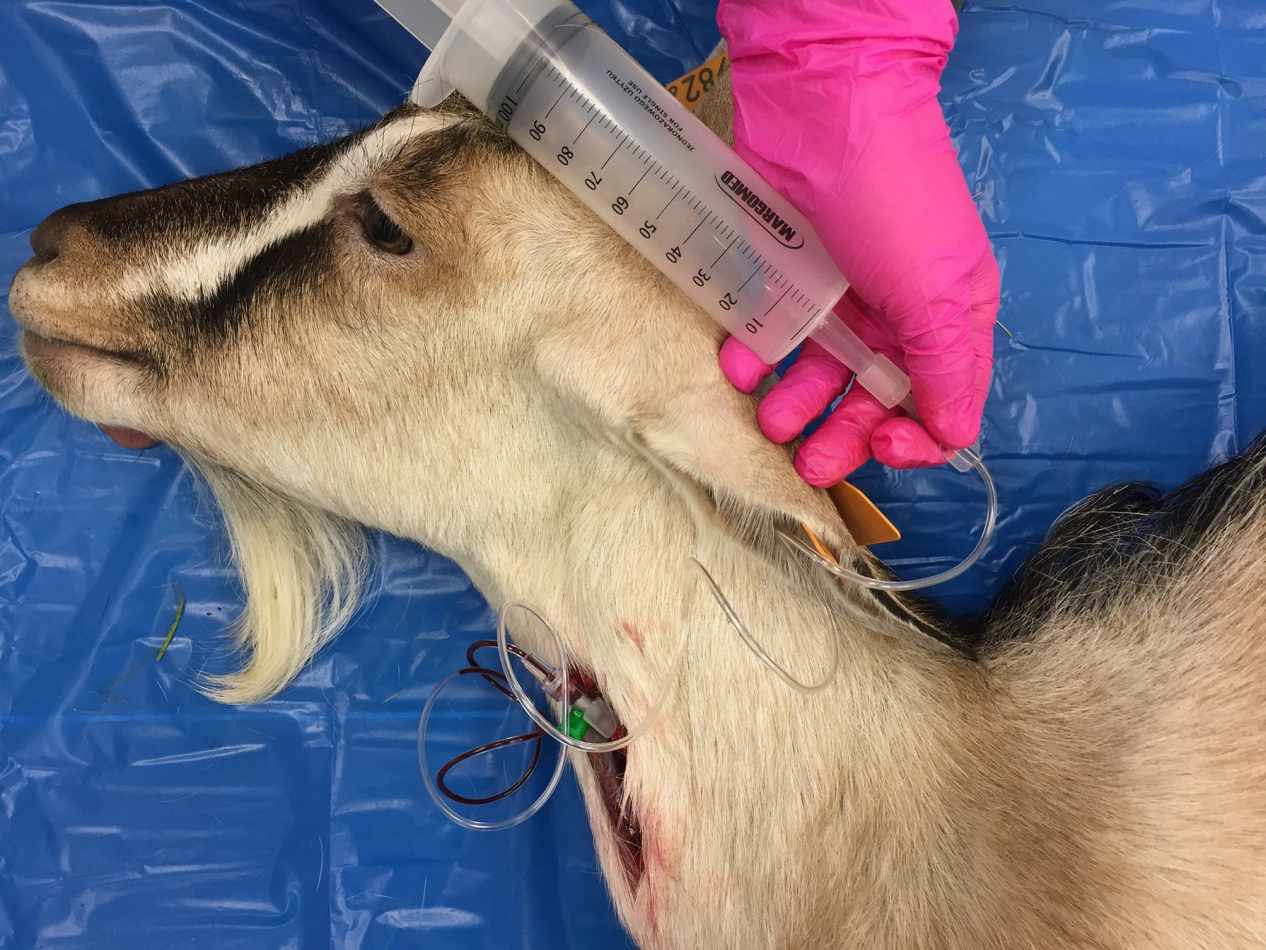
Air administration using a flexible plastic tubing and a 120 ml syringe.

The air was administered 10 to 300 minutes after death. Left or right common carotid artery was randomly chosen as access point to the vascular system. In goats, both left and right common carotid arteries branch together from bicarotid trunk, therefore we assumed no difference in filling degree regardless of the contrast administration side. Access to the artery was obtained after cutting the skin and subcutaneous tissues in the upper one third of the neck (Fig 1). After visualization of the artery the intravenous 18G catheter was placed, towards the trunk (Fig 2). Then, using a flexible plastic tubing, air was introduced manually by a 120 ml syringe (Fig 3).

**Fig 4:**
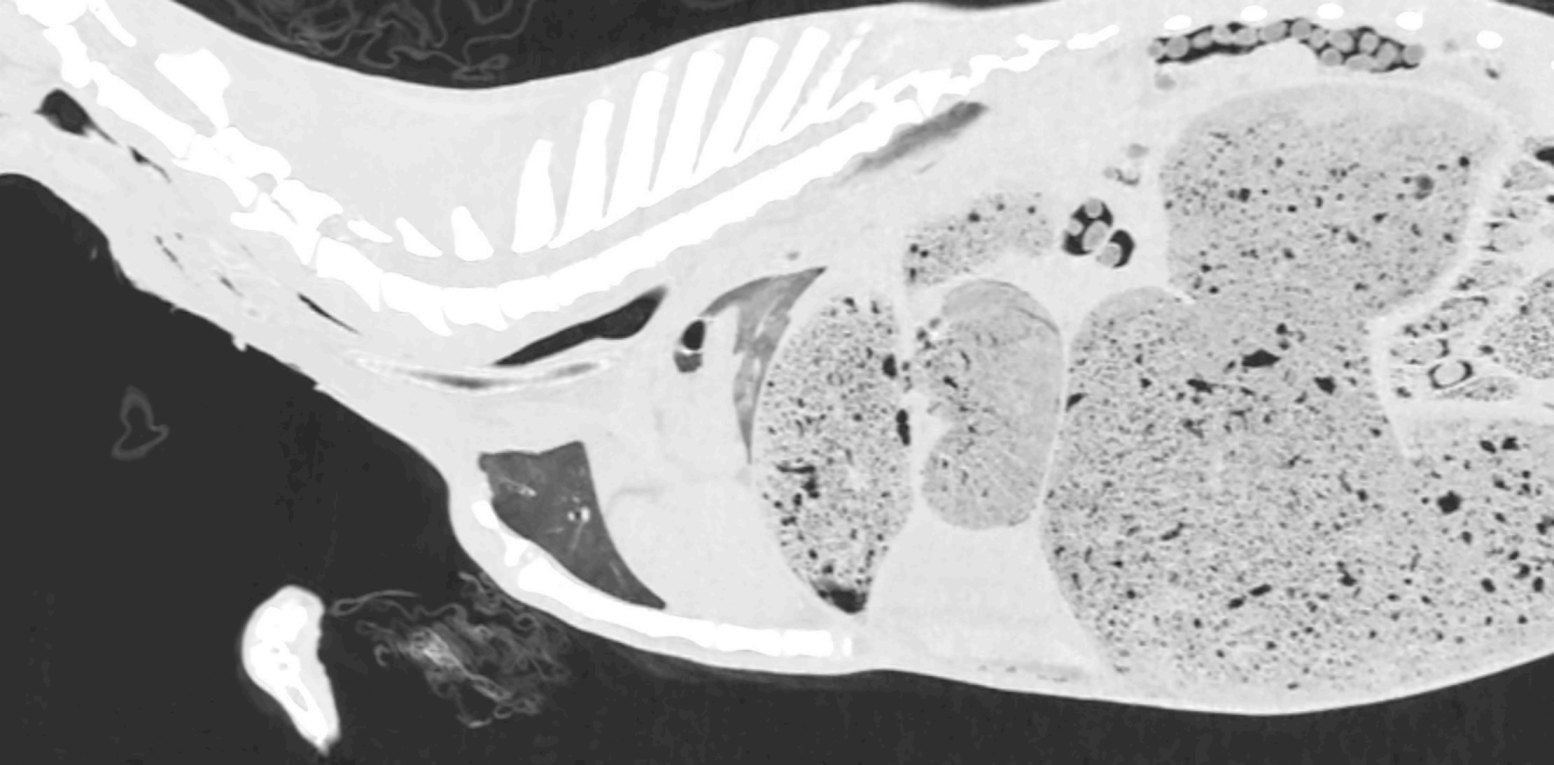
CT reconstruction in parasagittal plane before intra-arterial air inflation; lung window. Air visible only in the cervical and thoracic part of the esophagus and intestines.

**Fig 5:**
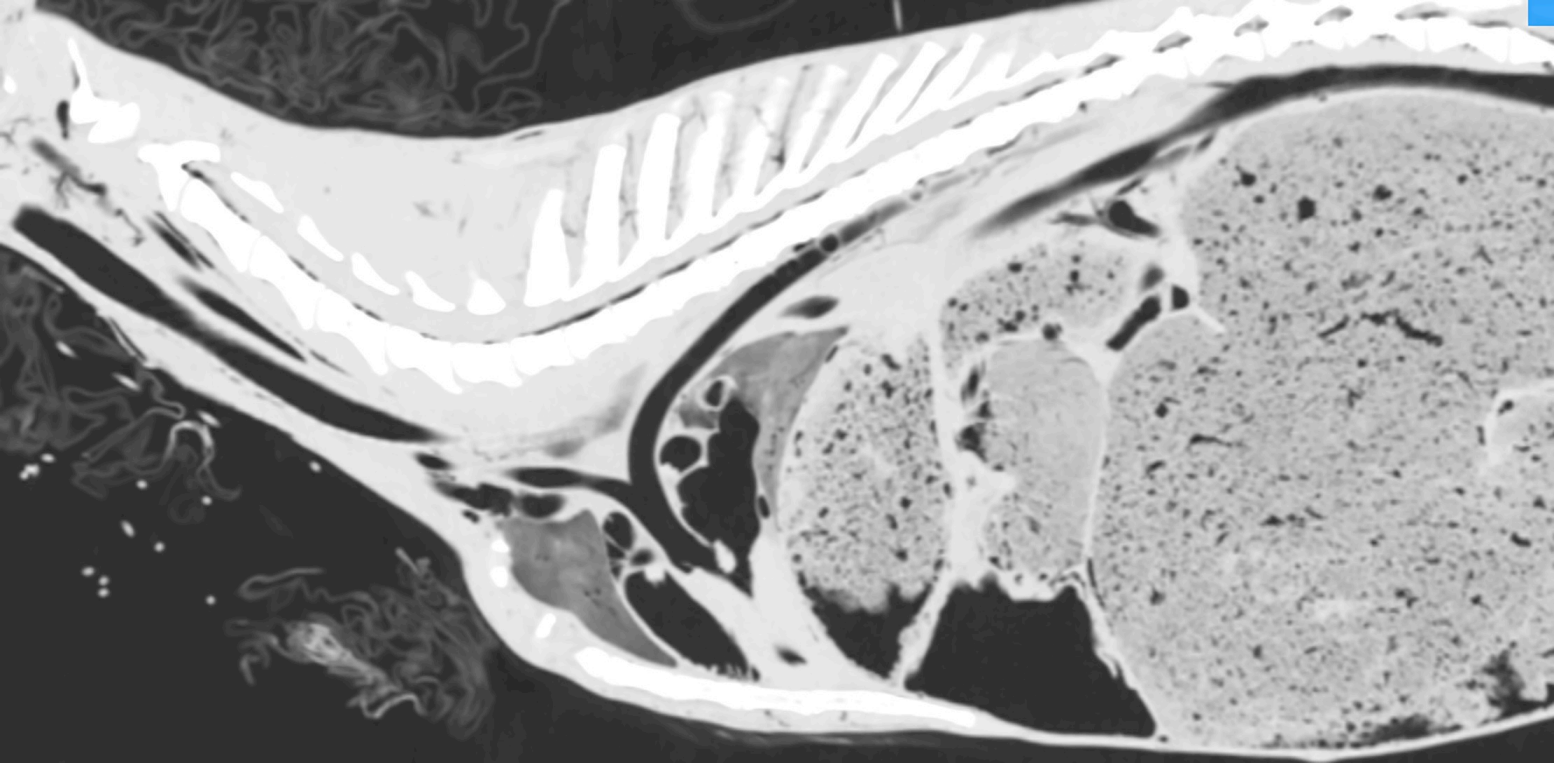
CT reconstruction in parasagittal plane after intra-arterial air inflation; lung window. Air-filled blood vessels and heart chambers visible.

**Fig 6:**
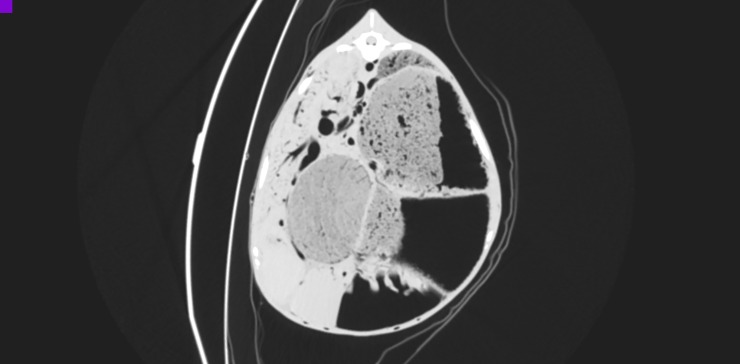
CT reconstruction in transverse plane at the level of the 12th thoracic vertebra after intra-arterial air inflation; lung window. Air-filled blood vessels visible, among others aorta, vena cava, portal vein, splenic veins, hepatic veins.

**Fig 7:**
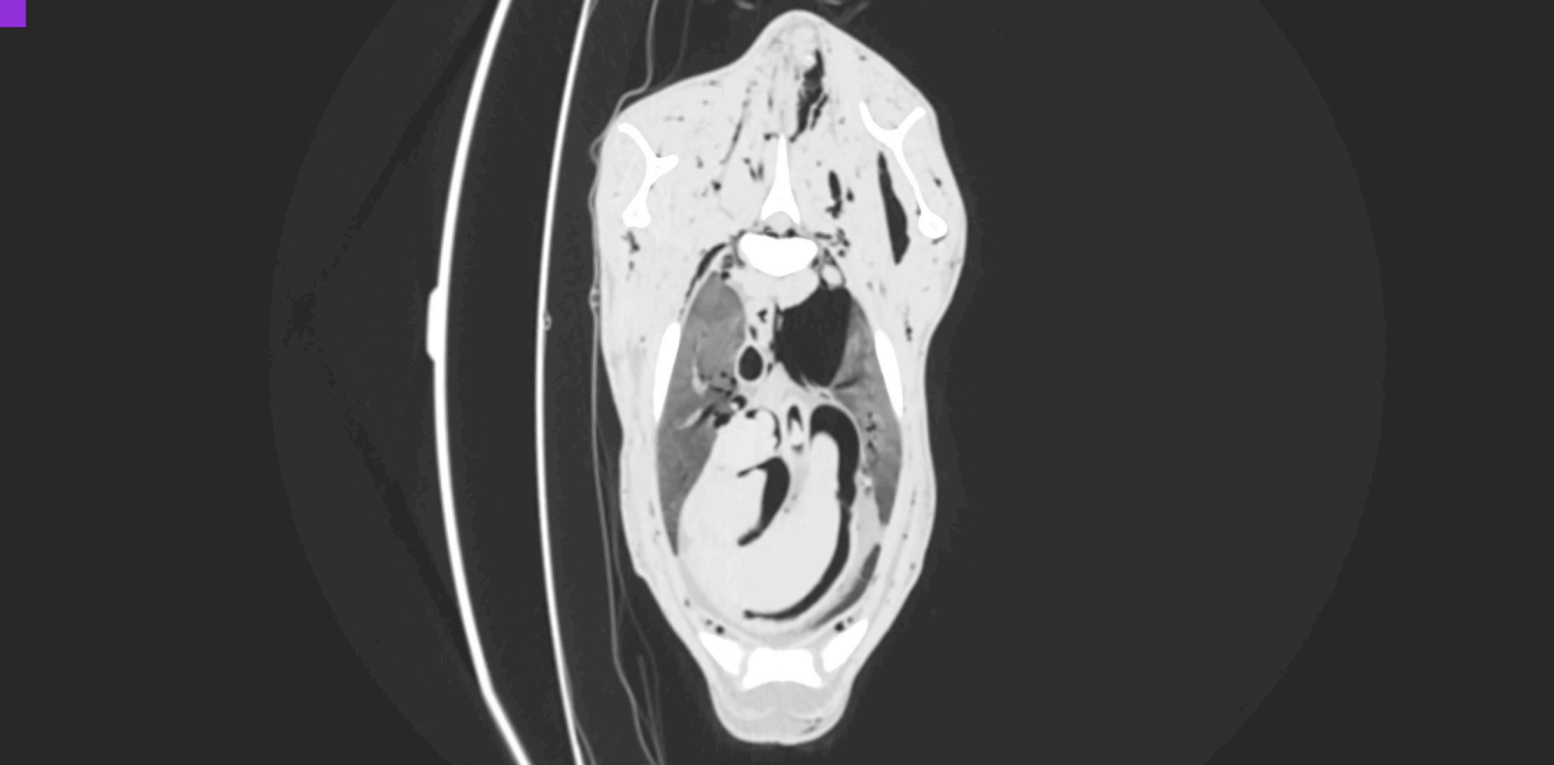
CT reconstruction in transverse plane at the level of the 3rd thoracic vertebra after intra-arterial air inflation; lung window. Air-filled blood vessels and heart chambers visible. Furthermore, subcutaneous emphysema in the region of withers and left pneumothorax due to air efflux from the vessels.

**Fig 8:**
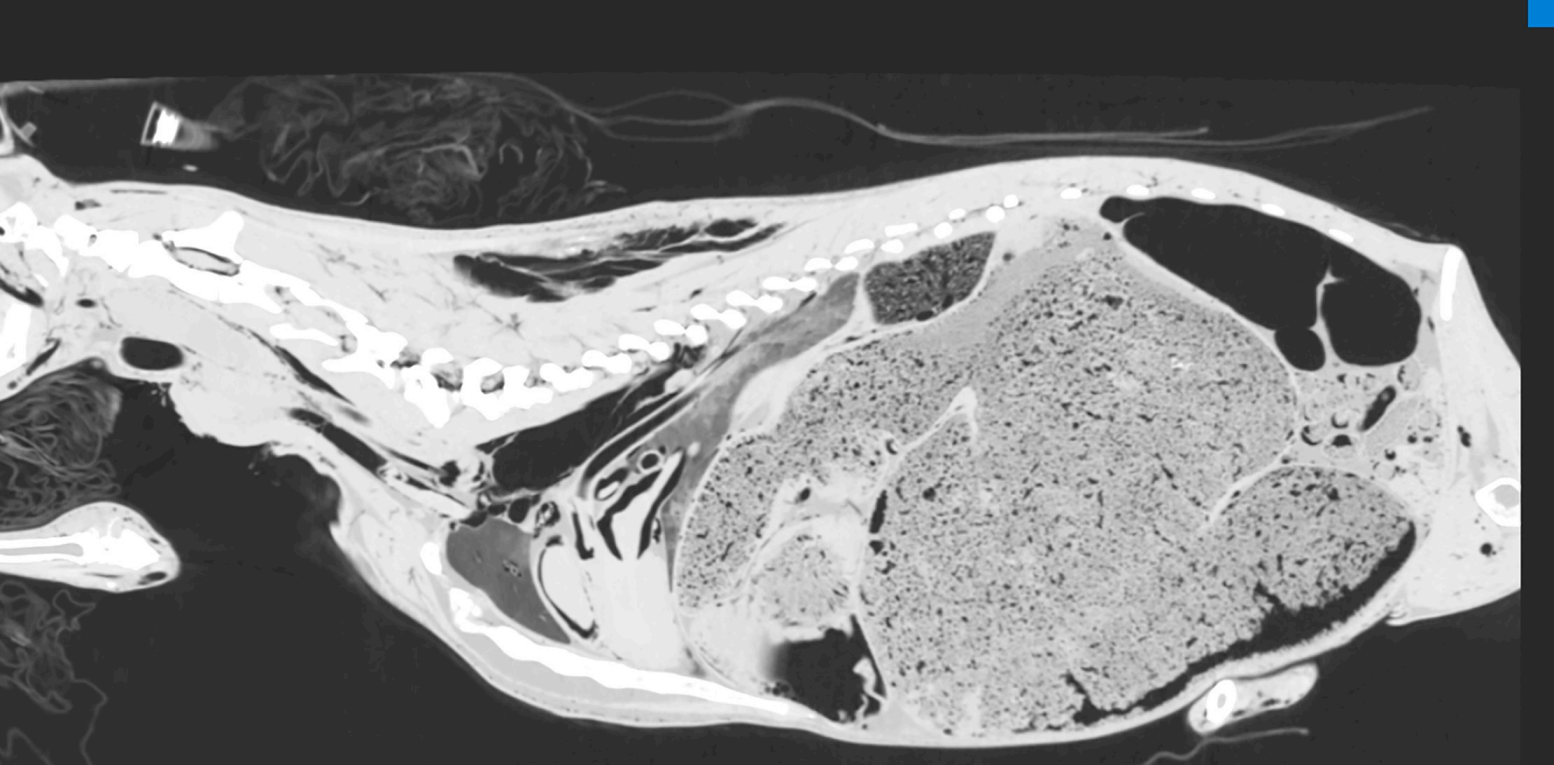
CT reconstruction in parasagittal plane after intra-arterial air inflation; lung window. Air-filled blood vessels and heart chambers visible. Furthermore, subcutaneous emphysema in the region of withers and neck, pneumothorax and extraperitoneal emphysema due to air efflux from the vessels.

CT scans were performed before and immediately after air administration, without changing the position of the animal (i.e. left or right recumbency). In 18 animals 16-slice helical scanner (Neusoft, NeuViz 16) was used with the parameters as follows: 120 kV, 200 mA, slice thickness 1–5 mm. In the remaining 15 goats 16-slice helical scanner (Toshiba, TSX-034A) with 1-mm-thick slices acquired at 120 V with automatic exposure control (AEC) (Figs 4–8).

### Data analysis

The quality of the CT scans was evaluated independently by two board-certified radiologists. A dedicated workstation (Osirix, Pixmeo, Geneva, Switzerland) was used.

### Statistical analysis

Blood vessel measurements were presented as the arithmetic mean ± standard deviation (SD), the median and interquartile range (IQR) and the range. Measurements of contralateral blood vessels were compared using the paired-sample Student’s t-test. The link between goats’ age and blood vessel measurements was investigated using the Pearson’s linear correlation coefficient (r). The Bonferroni correction of p-value (calculated as the primary p-value × the number of comparisons) was applied to all statistical tests to control for family-wise error. A significance level (α) was set at 0.05. Analysis was performed in TIBCO Statistica 13.3.0 (TIBCO Software Inc., Palo Alto, CA).

## Results

**Fig 9:**
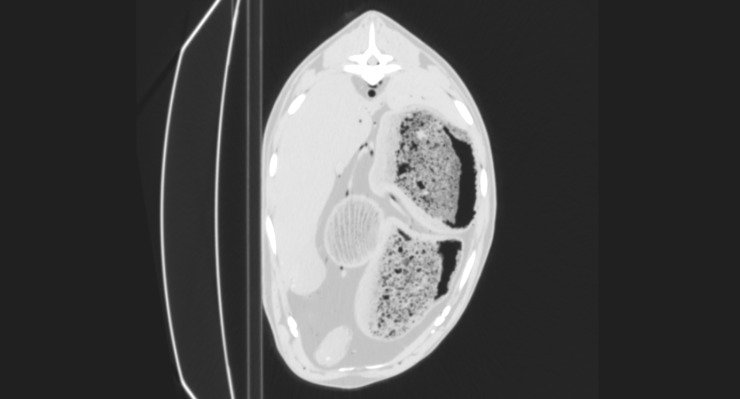
CT reconstruction in transverse plane at the level of the 12th thoracic vertebra after intra-arterial air inflation in volume 60ml/kg; lung window. Air-filled aorta is visible. Poorly air-filled liver and spleen veins. Complete lack of air-filling of the caudal vena cava and portal vein.

Initially, in 3 goats 60 ml/kg of air was administered and CT scans were performed. After administration of this amount of air only main vessels became visible (Fig 9) so an additional dose of 40 ml/kg of air was added. The volume of 100 ml/kg was still found as too little. After administration of further 20 ml/kg of air (a total dose of 120 ml/kg) all blood vessels in the entire body including head were filled and this dose was used in next 30 goats.

In all studied animals the vascular system filled with air was clearly visualized on CT scans. There were some differences in the degree to which blood vessels were filled with air. Main vessels, such as the ascending aorta, aortic arch, descending aorta, bicarotid trunk, brachiocephalic trunk, pulmonary trunk, cranial and caudal vena cava, were filled in all animals. In most of animals, pulmonary, hepatic, splenic, coronary and limb vessels were also visible. In some of them even small vessels from regions such as thoracic wall, vertebral canal, cranial cavity, mammary gland, kidneys and gastrointestinal tract were visible. Measurements of selected paired and unpaired blood vessels are given in the Tables 1 and 2, respectively. In 12 goats no or very few artefacts and in 11 goats some artefacts were visible. In remaining 10 goats many artefacts were present but blood vessels were still traceable. Vessel visualization was independent of the presence and the number of artefacts. In 29 goats (88%) this amount of air allowed to visualize vessels less than 4 mm in diameter. In 14 goats (42%) extravasation into tissues was observed, and 11 of them had also pneumothorax or pneumoperitoneum. During the autopsy performed after the CT examination, no significant changes related to the administration of the air contrast were noticed. The only observed changes i.e. blood foaming in the vessels during tissue cutting and the empty vessels seemed to be irrelevant and did not affect the histopathological examination. These results are consistent with the previous report[11]. There was no linear correlation between the age of goats and the blood vessel measurements (r from -0.35 to 0.55, all p-values >0.05).

**Table 1:** Descriptive statistics of paired blood vessels measurements (in mm) compared between sides using paired Student’s t-test with the Bonferroni correction of p-value (α = 0.05). ↑ indicates significant difference.

Blood vessel P	Left				Right				
	n of goats in which measurements were made	Mean ± SD	Median (IQR)	Range	n of goats in which measurements were made	Mean ± SD	Median (IQR)	Range	Student’s t-test p-value with the Bonferroni correction
**Arteriae**									
Common carotid artery	28	4.58 ± 0.75	4.5 (4.0–5.2)	2.9–5.8	27	4.51 ± 0.66	4.5 (4.1–5.1)	3.0–5.7	>0.999
Subclavian artery	33	4.89 ± 0.56	4.9 (4.6–5.4)	3.2–5.7	33	4.63 ± 0.69	4.5 (4.3–5.1)	2.9–5.8	0.309
Vertebral artery	28	4.17 ± 0.41	4.1 (4.0–4.4)	3.3–5.1	27	4.02 ± 0.56	3.9 (3.5–4.5)	2.5–4.9	>0.999
**Veins**									
External jugular vein		11.67 ± 1.54	11.2 (10.7–12.6)	9.9–15.8		11.82 ± 1.64	11.5 (11.0–12.9)	9.0–15.4	>0.999
Internal vertebral venous plexus	28	3.84 ± 0.68	3.8 (3.4–4.4)	2.5–5.6	28	3.85 ± 0.79	3.7 (3.2–4.3)	2.9–5.4	>0.999
Cavernous sinus	28	9.84 ± 1.31	9.7 (9.2–10.0)	6.8–12.8	28	9.72 ± 1.15	9.5 (9.2–10.2)	6.8–11.9	>0.999
Linguofacial vein	28	7.70 ± 1.07	7.6 (6.8–8.3)	6.1–11.1	26	7.21 ± 0.97	67.3 (6.4–8.0)	5.9–9.0	>0.999
Maxillary vein	28	↑9.21 ± 1.42	8.9 (8.1–10.3)	7.2–12.7	28	8.48 ± 1.22	8.5 (7.6–9.3)	6.3–11.9	0.031
Common iliac vein	28	↑8.15 ± 1.60	7.7 (7.5–8.0)	6.5–12.5	28	7.84 ± 1.59	7.6 (6.9–8.1)	5.7–11.8	0.581 0.047
Caudal lobar vein	33	10.21 ± 1.48	910.0 (8.9–11.0)	7.9–13.4	33	↑10.82 ±1.46	11.2 (9.4–12.1)	8.0–13.5	0.051

**Table 2:** Descriptive statistics of unpaired blood vessels measurements (in mm).

Blood vessels	n of goats in which measurements were made	Mean ± SD	Median (IQR)	Range
**Arteriae**				
Ascending aorta	32	111.96 ± 1.41	12.1 (11.1–12.8)	9.2–15.9
Descending aorta (at the level of aortic hiatus)	33	8.38 ± 0.79	8.3 (7.7–8.7)	7.2–10.0
Brachiocephalic trunk	33	9.16 ± 1.08	9.0 (8.4–9.9)	6.8–11.7
Celiac artery	29	5.61 ± 0.74	5.8 (4.9–6.2)	34.0–6.8
Cranial mesenteric artery	29	6.16 ± 0.87	6.4 (5.3–6.8)	4.4–7.5
**Veins**				
Dorsal sagittal sinus	28	3.26 ± 0.34	3.2 (3.0–3.5)	2.8–3.9
Cranial vena cava	32	15.81 ± 1.86	15.8 (14.1–17.1)	113.4–19.8
Caudal vena cava (at the level of 6^th^ thoracic vertebra)	32	16.57 ± 2.28	15.9 (15.7–17.5)	13.4–24.0
Caudal vena cava (at the level of 4^th^ lumbar vertebra)	29	14.02 ± 1.61	14.7 (13.1–15.1)	11.0–16.1
Left azygos vein (at the level of 4^th^ thoracic vertebra)	33	6.53 ± 1.61	6.0 (5.7–6.8)	3.7–11.0
Left azygos vein (at its origin at the level of the lumbar spine)	33	3.39 ± 0.55	3.3 (2.9–3.8)	2.7–4.9
Portal vein (the highest diameter)	28	18.65 ± 1.99	18.9 (17.3–19.9)	115.0–23.0
Splenic vein (at the splenic hilum)	28	7.97 ± 1.84	7.7 (6.9–9.3)	5.0–11.2
Right hepatic vein	32	5.98 ± 1.56	5.6 (4.8–6.7)	3.6–9.7

## Discussion

Post mortem CT has many advantages, however high costs and limited availability of the equipment render it a rarely employed diagnostic procedure. Oily contrast is expensive as is also a special pump which produces circulation. The use of air as a contrast medium significantly improves imaging quality and reduces costs. On the other hand owners are reluctant to give their consent to the post mortem examination because of a strong emotional connection with their animal. The proposal of the post-mortem examination involves a great deal of sadness, which is often the reason for the refusal. Similar situation is observed in case of humans, when the family finds the idea of their loved ones being dissected after death upsetting[12]. PMCTis an alternative to anatomopathological examination which may allow to establish definitive diagnosis in many situations. PMCT can reveal some causes of death, like massive bleeding, cardiac tamponade, massive pleural effusions and large pneumothorax. Compared to autopsy, PMCT can be reviewed easily and repeatedly by many professionals, since CT scans exported to DICOM files can be shared immediately all over the world[1]. The whole PMCT procedure including preparations is short, takes less than 20 minutes including CT scanning. The necessary equipment is very inexpensive (the cost is about 1 euro and can be used repeatedly). In addition, some protocols require three full exams so that the scans obtained are of sufficiently high quality[1]. This method allows to visualize the vascular system of the head, thorax, abdomen and limbs. It is important to emphasize that CT scans of the living animals vary from these obtained post mortem due to rigor mortis which changes the shape of organs. Moreover, the longer time elapses from death to CT examination, the more changes related to the body decay such as gas accumulation due to bacteria growth or migration of body fluids are visible. On the other hand, the main advantage of PMCT versus ante mortem examination is the lack of motion-related artefacts resulting from heartbeat and breathing.

Our study also has some limitations. All examinations were performed 10 to 300 minutes after death. Further research is needed to assess whether this method is useful for animals dead for longer time. However, in some human studies CT scans with air were performed up to 4 days after death and with oil contrasts even after a 4 month freezing period with good result[13]. Our study was performed on animals that were intended for culling due to low productivity or reduced welfare but none of them suffered from a disease which could affect their circulatory system or the integrity of blood vessels. Further investigations in animals with any morphological pathologies are needed.

In PMCT where oil contrasts are used, leakage into the perivascular space can be a disadvantage. Similarly, if water-soluble contrasts are used, edema around the vascular walls may be present. Such artefacts were present also in our study. We observed extravasation of air into the tissues in roughly the half of goats, however it did not affect visualization of vessels nor further anatomopathological examination. This finding is consistent with previous observations[11]. In this context, air as a negative contrast seems to be superior to liquid contrast agents. However, in our study blood frothing was observed, especially in the heart chambers. Such artefacts are not observed in case of liquid contrasts. Probably this could have been avoided by slower air injection.

In vast majority of goats the air reached also very small vessels, allowing for their precise visualization. As the air is very poorly soluble in the blood to fully fill all the vessels and heart cavities, blood must be pushed out to the surrounding tissues. In case of insufficient ejection, it becomes problematic to distinguish the vessel or heart wall from the adjacent blood as there is only a little difference between radiodensity of blood and soft tissues. As a consequence, the measurement of the thickness, for example, of the hearth muscle can be inaccurate. This problem does not occur in water-soluble contrast media, e.g. iodine, where there is a marked difference in blood and soft tissue boundaries.

Our study confirms earlier observations that air administration significantly improves the imaging of the organs, which can be recognized thanks to good visibility of the vessels[11].

Concluding, air appears to be an effective contrast agent for CT vascular imaging in veterinary forensic radiology. It is also much less expensive and readily available compared to special contrast agents.

## Supporting information

S1 Table Minimal data set. (XLSX)
